# Could SARS-CoV-2-Induced Hyperinflammation Magnify the Severity of Coronavirus Disease (CoViD-19) Leading to Acute Respiratory Distress Syndrome?

**DOI:** 10.3389/fimmu.2020.01206

**Published:** 2020-05-27

**Authors:** A. S. Smiline Girija, Esaki M. Shankar, Marie Larsson

**Affiliations:** ^1^Department of Microbiology, Saveetha Dental College and Hospitals, Chennai, India; ^2^Infection Biology, Department of Life Sciences, Central University of Tamil Nadu, Thiruvarur, India; ^3^Molecular Medicine and Virology, Department of Biomedicine and Clinical Sciences, Linköping University, Linköping, Sweden

**Keywords:** cytokine storm, corona virus disease 2019, IL-6, TNF-α, IL-1β

The exaggerated immune response induced in the lower respiratory tract against coronaviruses (CoVs), including CoViD-19 (2019-nCoV), appears to contribute to the overwhelming lung damage caused by the disease in comparison to the effect of the direct viral invasion and replication in the host. While it has resulted in high global rates of morbidity (4,618,821 infected cases), a sizeable number of individuals have already succumbed (311,847 deaths)^1^ (case fatality rate of 1–10%) to severe pathological manifestations involving the lower respiratory tract (1) as of May 18, 2020, as reported by the World Health Organization^1^. This has, however, been documented to be less severe when compared to influenza (2).

CoViD-19 reportedly has four stages: a pre-symptomatic phase of fever, cough, and generalized malaise heralded by high viral loads in severely affected cases. After about a week, the second stage manifests with viral pneumonia that involves the lower respiratory tract (while viral loads in the upper respiratory tract decrease exponentially). A vast majority of patients show clinical improvement as protective humoral responses are developed at this stage of the disease. A minor proportion of individuals progress to the third phase of CoViD-19 by developing symptoms of hypercytokinemia (cytokine release syndrome (CRS)/cytokine storm) characterized by exaggerated levels of pro-inflammatory cytokines and other pathognomonic biomarkers of inflammation, leading to the rapid onset of acute respiratory distress syndrome (ARDS) and multi-organ failure (Stage 4). It is also intriguing to know that many individuals with CoViD-19 have not developed ARDS. The median time from development of symptomatic disease to death from CoViD-19 is ~2–8 weeks (3). SARS-CoV-2 appears to trigger a prolonged phase of hypercytokinemia (also called as macrophage activation syndrome) that encompasses a broad array of pro-inflammatory mediators like IL-6, IL-1β, TNF-α, and CXCL8 (IL-8) together with the infiltration of inflammatory and degranulating cells into the lungs, usually 7–10 days following the onset of symptoms during the second stage of CoViD-19 (3–7). Variations in human genetic make-up have been shown to affect disease progression and prognosis of infectious diseases. A more recent emergence of interest surrounds individuals harboring mutations in the Mediterranean fever gene (*mefv*), which likely could predispose the onset of severe CoViD-19 disease manifestations resulting from local and systemic cytokine storm (8).

Cytokine storm refers to a systemic acute inflammatory manifestation triggered during viral infections characterized by an upsurge in immune cells and cytokine levels (9). It occurs when leukocytes become activated leading to an abrupt release of TNF-α, IL-6, IL-1β, and IL-10, which at times can be life-threatening due to the acute onset of hypotensive shock and multi-organ failure (9), as reported in CoViD-19^1^ (3). Cytokine storm likely could dampen innate and adaptive immune responses against SARS-CoV-2 infection. Cytokine storm pathophysiology in CoViD-19 is often reported to be due to high levels of IL-6 in individuals (9), although this, we believe, could synergize with TNF-α and IL-1β levels. A similar kind of hyperactive inflammatory response also appears to have occurred in SARS-CoV and MERS-CoV infections culminating in severe lung fibrosis, often with poor disease prognosis (10). Recent reports suggest that CoViD-19 disease is characterized by an exaggerated release of acute phase reactants that includes C-reactive protein (CRP), serum amyloid A, and ferritin, suggesting a rapid activation of the innate immune response (11, 12). Individuals with CoViD-19 reportedly possess elevated levels of circulating TNF-α, IL-1β, IL-1Rα, sIL-2Rα, IL-6, IL-10, IL-17, IL-18, IFN-γ, MCP-3, M-CSF, MIP-1α, G-CSF, IP-10, and MCP-1 (13). Reports suggest that IL-6, IL-8, and TNF-α attributes to SARS-related ARDS. Further, development of lung damage is likely due to the elevation of inflammatory cytokine levels and CRP in SARS patients. Importantly, high levels of serum TNF-α tends to be seen more prevalently in patients who die of SARS-CoV-1 than in those who survive (14). However, emerging reports of SARS-CoV-2 suggests the predominance of IL-6 over TNF-α although this is yet to be confirmed from multiple findings (15).

IL-6 is predominantly produced by lung epithelial cells in response to stimulatory factors similar to what has been shown for several other respiratory viruses, including SARS-CoV and MERS-CoV. IL-6 is produced in a constitutive manner only upon stimuli and not by resident immune cells of the lungs, thus portraying its pleotropic and immuno-regulatory role in the respiratory mucosa. Although IL-6 is regarded as a marker of pneumonia in CoV infections, it has now become evident that abrupt release of IL-1β and TNF-α could contribute to the severity of CoViD-19 pathogenesis. The onset of cytokine storm in the lungs likely occurs prior to the recruitment of inflammatory cells, especially in allergic patients and those with other co-morbidities, leading to an exorbitant rise in mortality rates (16). A similar cytokine storm that led to severe lung injury resulting from the release of 18 inflammatory mediators has been demonstrated in SARS-CoV-infected patients (17). Immune-mediated damage to the lungs and other organs, and subsequent development of multi-organ dysfunction, is explained by hypercytokinemia resulting from cytokine release largely by SARS-CoV-infected ACE2-expressing cells, but not by uninfected cells (18). More recent experimental investigation has reported dramatically high levels of CXCL10, CCL5, and IL-1β in human lung epithelial cells and in the lung tissues of SARS-CoV-infected mice. The report has established that pulmonary inflammation was modulated via NLRP3, providing key clues to the development of potential antiviral targets (19).

It has also been reported that individuals admitted into intensive care units have significantly elevated levels of IL-6, IL-10, and TNF-α and fewer T cells in circulation (20). Interestingly, it has also been reported that CoViD-19 disease severity correlates positively with a concomitant rise in inflammatory cytokine levels that also drives the depletion and exhaustion of SARS-CoV-2-specific CD8+ T cells (20). It has also become evident that the frequency of circulating CD4+ and CD8+ T cells are exponentially reduced and show signs of hyperactivation, i.e., an elevated expression of HLA-DR and CD38. Interestingly, the hyperactive CD8+ T cells were also enriched with perforin and granulysin that potentially adds to the reported lung injury (21). More recent findings point to the consistently elevated levels of CXCL10, CCL7, and IL-1 receptor antagonist and their association with an increased viral load, exacerbated lung injury, and a fatal prognosis.

Published data from SARS-CoV-infected patients points to an increase in inuf6 TNF-α levels, enhancing the migration of inflammatory cells viz. eosinophils and neutrophils (22). A cohort of 41 laboratory-confirmed CoViD-19 patients in Wuhan, China, subjected to serological evaluation, revealed high levels of IL-1β, IFN-γ, IP-10, and MCP-1, of all the 22 cytokines tested among both ICU as well as non-ICU cases. It has also become evident that in moribund cases, cytokine storm was highly associated with the magnitude of disease severity (12). Subsequent experimental data also suggests that production of TNF-α is mediated via NF-κB through the degradation of I-κBα by CoV spike proteins (23). CoVs being predominantly zoonotic, a similar up-regulation of TNF-α has also been documented in feline CoV infection (24).

More recently, the direct involvement of the NOD-like receptor family protein (NLRp-3) inflammasome has come to light in SARS-CoV 3a culminating in the release of IL-1β via ion channel proteins called viroporins (25). In addition to the classical cytokine storm, CCL2, CXCL10, CXCL9, and CXCL8 upregulation has also been reported in uncomplicated SARS-CoV infections (25). The underlying rationale behind the far-reaching prognosis of CoViD-19 in Wuhan, China, is believed to involve virus-activated cytokine storm syndrome or fulminant myocarditis, which could be related to secondary haemo-phagocytic lympho-histiocytosis (sHLH), an under-recognized ailment most commonly triggered by viral infections and sepsis, and is co-related with CoViD-19 disease (1).

The proposed cytokine storm in the pathogenesis of CoV could result in deleterious consequences with varying degrees of immunopathology (Figure 1). As an initial step, infiltration of the airway by IFN-αβ and IFN-μ mediated by Fas-FasL-/TRAIL-DR5-dependent mechanisms leads to endothelial cell apoptosis and vascular leakage, which will be followed by TNF-mediated T-cell apoptosis resulting in suboptimal responses of T cells. Through the abrogation of STAT-1 signaling specifically in myeloid cells, activated macrophages can accumulate and alter the homeostasis of lung tissue. The final phase of the cytokine assault by IL-6, CXCL8, IL-1β, and GM-CSF, CCL2, CCL5, IP-10, and CCL3 reportedly results in ARDS (26).

**Figure 1:**
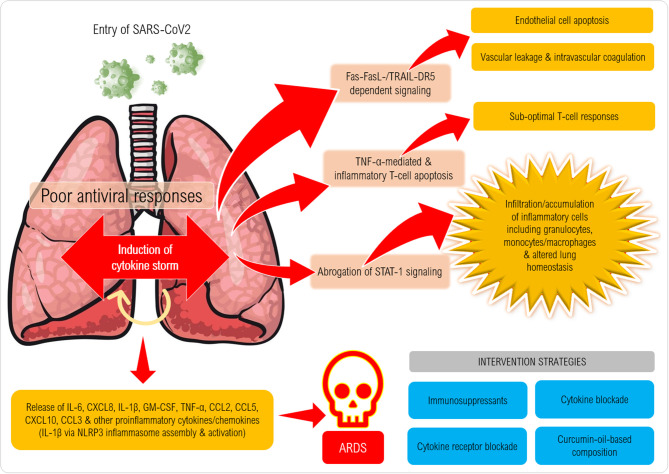
Proposed mechanism of induction of cytokine storm in coronavirus disease (CoViD-19). Following entry of SARS-CoV-2 into a susceptible host, the virus employs its spike protein to invade the respiratory airway epithelial cells via ACE2 receptors expressed on the cells causing damage to the upper respiratory epithelium. Several danger-associated molecular patterns, cellular stress factors (IL-1a, IL-33, HMGB1) and pro-inflammatory chemokines and chemoattractants (eg., CXCL8, CXCL10, C3a, C5a) are released that recruit several types of inflammatory cells (monocytes/macrophages, granulocytes and NK cells) that release IL-1β, IL-6, IL-18, TNF-α, IFN-γ and several other factors that can further trigger inflammation (also via NLRP3 and AIM2 inflammasome assembly and caspase-1 activation) especially in the lower respiratory tract. Mast cell, macrophage and endothelial activation also takes place to exaggerate the inflammatory cascade resulting in cytokine storm syndrome (or hypercytokinemia). Excessive cytokine release and binding to cytokine receptors lead to massive cytokine signaling that culminates in Fas-FasL/TRAIL-DR5-dependent signaling in endothelial cells causing their death, which erodes the blood vessel walls that results in vascular leakage. Intravascular coagulation also ensues leading to widespread damage of blood capillaries in the lungs. T cell death/depletion ensues via TNF-α and also expression of exhaustion molecules (PD-1) on CD4+ and CD8+ T cells (not shown) can result in poor anti-viral immune responses. Onset of acute respiratory distress syndrome can be fatal characterized by pneumonitis, pyrexia, myalgia, dyspnoea, loss of smell/taste and can lead to high mortality rates.

It must be considered that an ongoing phase of immunosenescence in the mucosa of elderly individuals deteriorates CoV severity, leading to poor levels of functional T-cell subsets, antigen-specific IgA, and immunological remodeling. CoV also display neuro-virulence attributes, differentially inducing the production of pro-inflammatory mediators by astrocytes and microglial cells, as shown in experimental mice (27). Intriguingly, the onset of cytokine storm in CoViD-19 disease can be hypothesized to be gender-biased, as the closely related MERS-CoV infection exhibited a higher incidence in males than females (28). Gender-based variations in the expression of ACE2 could likely have implications in severe disease progression resulting from cytokine storm. Coding variants at specific amino acid sites are likely to be a genetic risk factor for the development of severe CoViD-19 and could affect human males and females differently. Surveys conducted on the follow-up of patients with SARS-CoV suggest a strong role for the involvement of cytokine storm (29).

Together, to control the askew and flared cytokine assault, and to likely alleviate lung pathology and increased survival rates, the efficacy of immuno-suppressants like actemra and IL-1β antagonists like anakinra could be investigated. Tocilizumab (a recombinant humanized anti-human IL-6 receptor monoclonal antibody) specifically binds sIL-6R and mIL-6R to inhibit signal transduction and has been well-tolerated as established in animal drug trials (30, 31). A recently published CoViD-19 research has shown encouraging results with no evidence of any serious adverse events (32). A multicentric randomized-controlled trial of tocilizumab has been approved for CoViD-19 pneumonia (ChiCTR2000029765) (33). Application of artificial liver purification systems in addition to the rapid detection of cytokine index should be considered for implementation. Recently, an *in silico* docking analysis has documented how curcumin, a known anti-inflammatory blockade strategy, can potentially inhibit the main protease (M-Pro) of CoViD-19 (34). The importance of studies on the association between specific HLA loci/haplotypes, genetic predispositions, and the development of anti-SARS-CoV-2 immune responses also is urgently warranted. As a measure of restraint, it is indeed the need of the hour to discover or repurpose improved concepts for disease control as well as for alleviating the magnitude of cytokine storm syndrome in the ongoing CoViD-19 pandemic.

## Author Contributions

AG: conception or design of the work, the acquisition, analysis or interpretation of data for the work, drafting the work or revising it critically for important intellectual content, provide approval for publication of the content, and agreed to be accountable for all aspects of the work in ensuring that questions related to the accuracy or integrity of any part of the work are appropriately investigated and resolved. ES: conception or design of the work, the acquisition, analysis or interpretation of data for the work, provide approval for publication of the content, and agreed to be accountable for all aspects of the work in ensuring that questions related to the accuracy or integrity of any part of the work are appropriately investigated and resolved. ML: provided approval for publication of the content and agreed to be accountable for all aspects of the work in ensuring that questions related to the accuracy or integrity of any part of the work are appropriately investigated and resolved.

## Conflict of Interest

ES is the Associate Editor of Frontiers in Immunology. The remaining authors declare that the research was conducted in the absence of any commercial or financial relationships that could be construed as a potential conflict of interest.

## Footnotes

^1^https://www.who.int/emergencies/diseases/novel-coronavirus-2019/situation-reports/
